# Acute and Subclinical Myocardial Injury in COVID-19

**DOI:** 10.14797/mdcvj.1038

**Published:** 2021-12-15

**Authors:** Valentina L. Crudo, Ahmed I. Ahmed, Eilidh L. Cowan, Dipan J. Shah, Mouaz H. Al-Mallah, Maan Malahfji

**Affiliations:** 1 Houston Methodist DeBakey Heart & Vascular Center, Houston Methodist Hospital, Houston, Texas, US

**Keywords:** COVID-19, coronavirus, cardiovascular disease, myocardial injury, myocarditis

## Abstract

Coronavirus disease 2019 (COVID-19) is a global pandemic that, at the time of this writing, has led to 178,000,000 cases worldwide and more than 3,875,000 deaths. Cardiovascular complications of COVID-19 have become the focus of investigation after many hospitalized COVID-19 patients—with or without established cardiovascular disease—incurred clinical or subclinical myocardial injury, including isolated biomarker elevations, myocardial infarction, arrhythmia, heart failure, myocarditis, and cardiogenic shock. In this review, we highlight the most recent evidence of the prevalence and potential etiologies of acute and subclinical myocardial injury in COVID-19 patients.

## Introduction

For the last 2 years, the world has been facing a pandemic caused by severe acute respiratory syndrome coronavirus 2 (SARS-CoV-2), the causative agent of coronavirus disease 2019 (COVID-19). As of December 1, 2021, there were over 263 million cases of COVID-19 globally and roughly 5,223,470 COVID-19 related deaths, 782,056 in the United States alone.^1^ While most patients recover after experiencing mild or moderate symptoms, some patients, particularly those with risk factors and underlying comorbid conditions, can have severe manifestations and acute respiratory distress syndrome (ARDS).^2^

In the early phases of the pandemic, it was recognized that the virus leads to systemic disease, and cardiac, vascular, renal, and various organ system manifestations were reported.^3,4,5^ Indeed, myocardial injury in hospitalized COVID-19 patients has been observed in patients with and without established cardiovascular disease (CVD). Cardiac involvement includes isolated biomarker elevations such as troponin and brain natriuretic peptide, myocardial infarction, arrhythmia, myocardial inflammation and myocarditis, and thromboembolic events as well as heart failure and cardiogenic shock.^6^ The prevalence and short-term implications of myocardial injury in hospitalized COVID-19 patients have been investigated, although many questions remain regarding its mechanisms, optimal treatment strategies, and long-term consequences for cardiovascular health after recovery. In nonhospitalized COVID-19 patients and particularly those without known CVD, the incidence and prognosis of subclinical myocardial injury remains an active area of investigation with critical public health consequences for the long-term management of recovered patients. In this review, we discuss the current knowledge about the prevalence and various manifestations of acute and subclinical myocardial injury in COVID-19 patients.

## Sars-Cov-2 and Cardiovascular Risk Factors

SARS-CoV-2 is a single-stranded RNA virus that exerts its infectious action by coupling a spike protein (S-protein) with the body’s angiotensin-converting enzyme 2 (ACE2) receptor, which is expressed mainly in the lungs.^7^ ACE2 is also present in high concentrations in the heart, which may partly explain myocardial injury in COVID-19.^8^ Other viral illnesses, including H1N1 influenza, severe acute respiratory syndrome virus (SARS), and Middle East respiratory syndrome virus (MERS), have all been associated with cardiac injury including myocarditis.^9,10,11^

**Figure 1:**
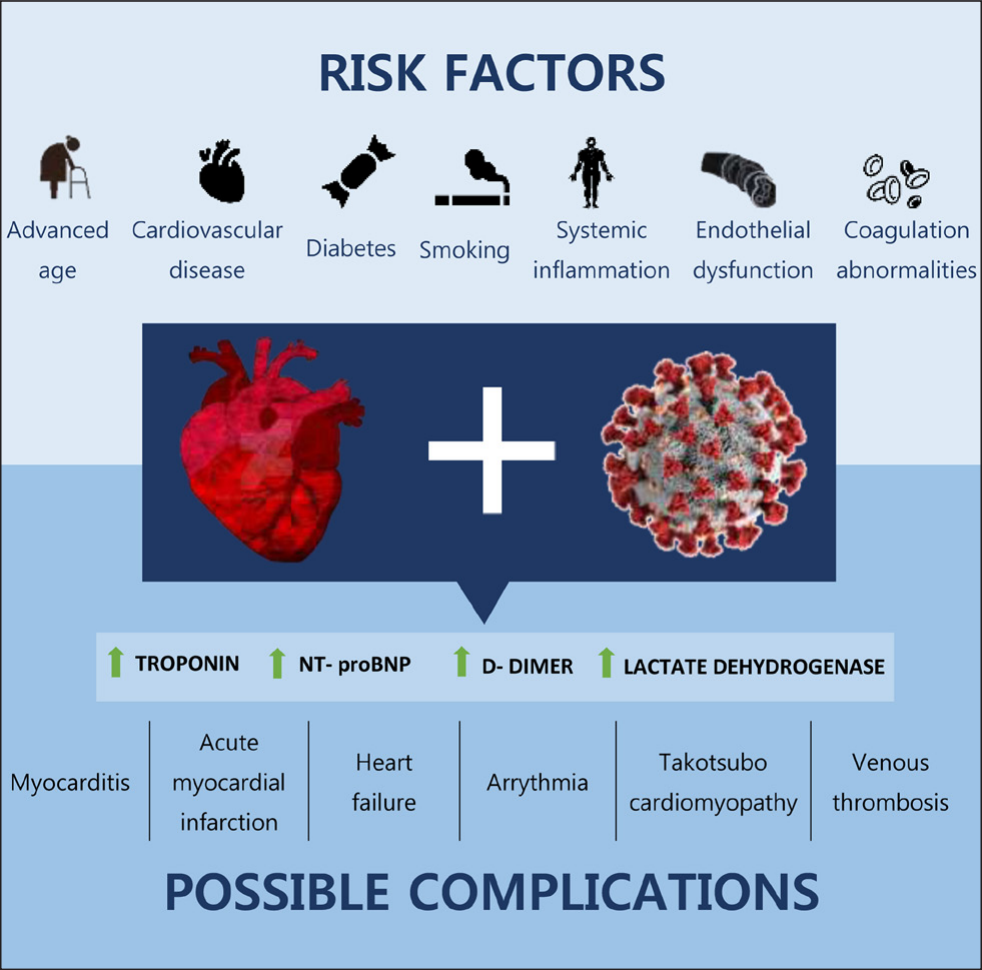
Risk factors for cardiovascular involvement of COVID-19 and possible complications.

Cardiovascular risk factors, mainly hypertension, obesity, and diabetes, are associated with a higher risk of hospitalization due to COVID-19.^12^ A meta-analysis of 13 studies with 3,207 total patients showed that the risk factors associated with disease progression and morbidity are age > 65 years, male gender, history of smoking, hypertension, preexisting cardiovascular disease (CVD), and respiratory disease (***Figure 1***).^13^ In addition, obesity has long been associated with worse clinical outcomes in viral infections.^14^ An analysis of the American Heart Association COVID-19 CVD registry revealed that all classes of obesity were associated with a progressively higher risk of COVID-19 complications, such as mechanical ventilation and in-hospital death. Obese patients with COVID-19 were likely to be admitted to the hospital at a younger age than non-obese patients and have a higher risk of venous thromboembolism and major cardiovascular events.^15^

### Biomarkers of Myocardial Injury

Studies have shown a prevalence of acute myocardial injury (as defined by troponin elevation) in hospitalized COVID-19 patients ranging from approximately 12% in one series to as high as 62% in another (***Table 1***).^12,16,17,18,19,20,21,22,23,24,25,26^ A large series from New York showed a prevalence of acute cardiac injury of 22.6% by a variety of troponin assays.^12^ Acute myocardial injury in COVID-19 is associated with in-hospital mortality. In a cohort study of 416 hospitalized COVID-19 patients, 19.7% had evidence of myocardial injury manifested by elevation of high-sensitivity troponin I levels and NT-terminal pro-B-type natriuretic peptide (NT-proBNP). These patients had a markedly higher in-hospital mortality rate (51.2%) compared with those without myocardial injury (4.5%). Furthermore, among those with myocardial injury, greater degrees of troponin elevation were associated with higher mortality rates.^19^

**Table 1:** Summary of cardiac injury prevalence (defined as troponin elevation) in COVID-19 studies.^12,18,19,20,21,22,23,24,25,26^ ICU: intensive care unit.


AUTHOR	*N*	(%) WITH ELEVATED TROPONIN	ASSAY USED	PATIENT SETTING

Metkus et al.^20^	243	51	Troponin I or T	ICU

Giustino et al.^21^	305	62	Troponin T	Inpatient

Huang et al.^22^	41	12	Hypersensitive troponin I	Inpatient

Han et al.^23^	273	5.05% (outpatients) 23.33% (inpatients) 20% (ICU)	Hypersensitive troponin I	Outpatient, inpatient, ICU

Richardson et al.^12^	5,700	22.6	Variety of assays	Inpatient

Petrilli et al.^24^	4,103	11.7	Not reported	Outpatient and inpatient

Wang et al.^25^	138	7.2	Troponin I	Inpatient

Zhou et al.^26^	191	17	High-sensitivity troponin I	Inpatient

Guo et al.^18^	187	27.8	Troponin T	Inpatient

Shi et al.^19^	416	19.7	High-sensitivity troponin I	Inpatient


It is unclear if COVID-19–related acute myocardial injury differs in prevalence in other viral infections. In a study of 1,131 patients with lab-confirmed influenza infection, only 33 (2.9%) had myocardial injury.^27^ In a study comparing 243 intubated COVID-19 patients with 506 ARDS patients without COVID-19, there was no statistically significant difference in cardiac biomarkers levels between the two groups, although troponin values were associated with worse outcomes in both groups.^20^

## Myocardial Inflammation and Myocarditis

Reports of myocarditis associated with coronavirus infection date back to the 1980s.^28^ Myocarditis is often a clinical diagnosis made without histopathologic confirmation because of poor utilization and sensitivity of endomyocardial biopsies.^29^ Myocardial inflammation associated with SARS-COV-2 became a focus of research studies early in the pandemic, although isolation protocols prohibited myocardial biopsy in most cases. Although early case reports localized viral particles in the myocardial tissue associated with low-grade inflammation,^30^ there is little evidence supporting direct cardiomyocyte injury through virus-mediated lysis. Histopathologic studies of myocardial biopsies or postmortem examinations have largely suggested that SARS-COV-2 myocarditis is uncommon or rare.^30,31,32,33^ Conversely, at least one acute histopathologic finding such as macro- or microvascular thrombi, inflammation, or intraluminal megakaryocytes was reported in almost half of postmortem cases.^31^ While acute and subclinical myocardial injury is not uncommon in COVID-19 cases, it appears that COVID-19–related nonischemic injury and myocardial inflammation have different mechanisms than in lymphocytic myocarditis.

## Role of Cardiac Magnetic Resonance

Cardiac magnetic resonance (CMR) is the standard noninvasive imaging modality for myocardial tissue characterization, and diagnosis of myocarditis by CMR has established criteria.^34^ However, diagnosis by CMR carries some limitations, such as abnormalities on T1/T2 mapping that are somewhat vague. Late gadolinium enhancement (LGE) can indicate acute injury or myocardial replacement fibrosis, and the pattern and context sheds light on the diagnosis in the right clinical setting.

**Figure 2:**
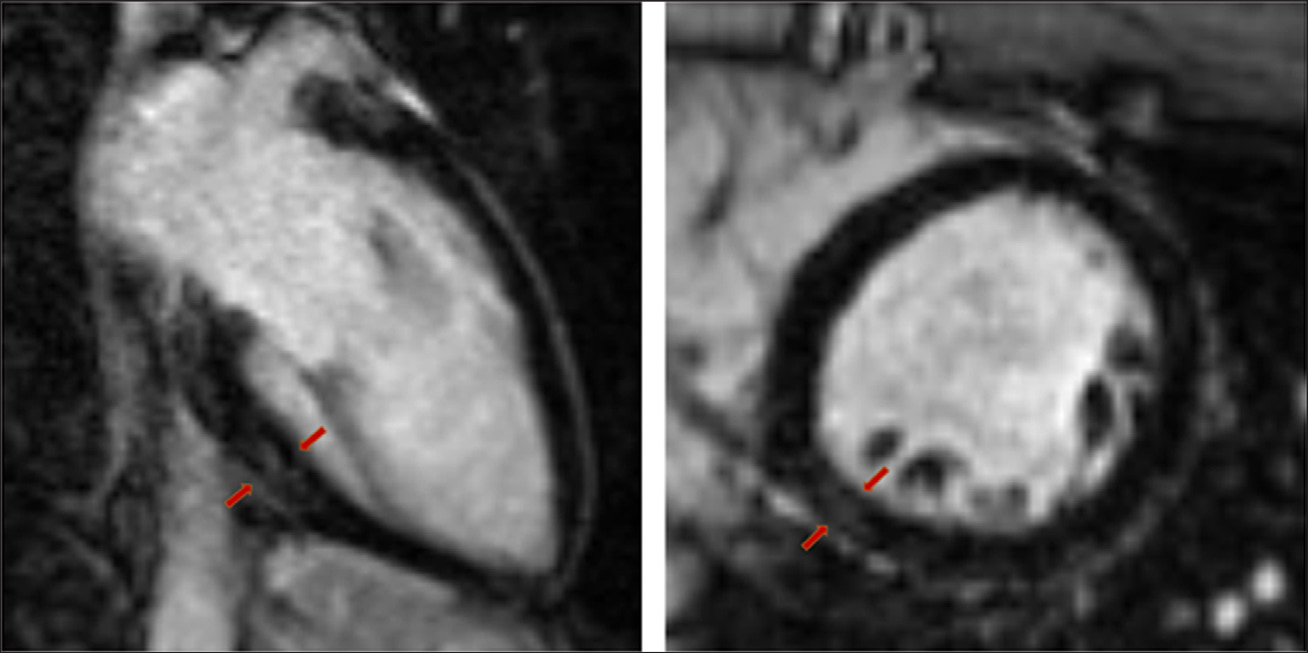
Cardiac magnetic resonance image of 25-year-old healthy male complaining of persistent chest pain and palpitations 5 weeks after COVID-19 diagnosis. Mild late-gadolinium enhancement can be visualized in the basal inferior and inferolateral myocardial wall. Holter monitoring showed frequent premature ventricular beats.

CMR-based research studies in COVID-19 focus predominately on patients who recovered after hospitalization. Studies have varied significantly thus far in the incidence of cardiac involvement, likely due to differences in the tested populations and, more importantly, the criteria used to define abnormalities (***Table 2***).^35,36,37,38,39,40,41,42,43,44^ An early retrospective CMR study of 26 recovered COVID-19 patients with cardiac symptoms showed that 58% had abnormal CMR findings, including abnormal global native T1 and T2, LGE, and impaired right ventricular function (***Figure 2***).^35^ Later, a prospective cohort study by Puntmann et al. of 100 patients who recovered from COVID-19 found a 78% prevalence of cardiac involvement and 60% prevalence of ongoing myocardial inflammation based on CMR abnormalities detected by native T1/T2 mapping, LGE, and pericardial enhancement. High-sensitivity troponin T was detectable in 71% of patients and significantly elevated in 5%, showing evidence of subclinical myocardial involvement.^36^ Raman et al., who compared 58 recovered COVID-19 patients after hospital discharge with matched controls using multiorgan magnetic resonance imaging and functional assessment, found a 26% rate of elevated T1 time in the COVID-19 cohort but no significant difference in T2 time or extracellular volume fraction against controls.^37^

**Table 2:** Main findings of studies utilizing CMR in COVID-19 patients.^35,36,37,38,39,40,41,42,43,44^ ECV: extracellular volume fraction; CMR: cardiac magnetic resonance; LGE: late-gadolinium enhancement; hsTNT: high-sensitivity troponin T.


FIRST AUTHOR, YEAR	COUNTRY	TYPE OF STUDY	NO. OF PATIENTS	RESULTS	PRIMARY END POINTS	CONCLUSION

Joy^38^ 2021	UK	Prospective blind study	74 recovered patients with mild cases vs. 75 controls	No difference in end points between recovered patients and controls	Cardiac involvement 6 months after recovery from mild COVID-19	Mild COVID-19 in healthy patients does not result in cardiovascular abnormalities.

Raman^37^ 2021	UK	Observational cohort study	58 recovered patients from moderate-severe COVID-19 vs. 30 matched controls	• 26% elevated basal myocardial T1 • No statistical difference in T2 and ECV	Cardiac involvement 2-3 months after recovery from COVID-19	Multiorgan inflammation persists after recovery from moderate-severe COVID-19.

Starekova^39^ 2021	USA	Case series	145 competitive athletes recovering from mild-moderate COVID-19 who underwent CMR 15 days after diagnosis	1.4% had CMR findings consistent with myocarditis	Prevalence of myocardial involvement in competitive athletes recovering from COVID-19	There is low prevalence of myocarditis in this population.

Daniels^40^ 2021	USA	Case series	1,597 competitive athletes recovering from COVID-19	2.3% had CMR-diagnosed myocarditis, clinical and subclinical	Prevalence of myocarditis in competitive athletes recovering from COVID-19	CMR screening in athletes recovering from COVID-19 should be considered for safe return to play.

Martinez^41^ 2021	USA	Cross-sectional study	789 professional athletes with COVID-19 infection, irrespective of symptoms	0.6% had CMR findings suggesting inflammatory heart disease	Prevalence of detectable inflammatory heart disease in professional athletes with prior COVID-19 infection	Few cases of inflammatory heart disease have been detected; safe return to play has been achieved.

Kotecha^42^ 2021	UK	Retrospective study	148 patients with severe COVID-19 requiring hospitalization	54% had LGE: • 26% myocarditis • 22% ischemia • 6% dual pathology	Assess myocardial injury in hospitalized COVID-19 patients after recovery	During recovery from severe COVID-19, myocarditis-like injury can be detected. Its functional consequence is not clear.

Puntmann^36^ 2020	Germany	Prospective observational cohort study	100 recovered patients vs. 107 controls	• Abnormal CMR findings in 78% of recovered COVID-19 patients • 73% raised myocardial native T1 • 60% raised myocardial native T2 • 32% LGE • 22% pericardial involvement	Cardiac involvement after recovery from COVID-19	CMR revealed cardiac involvement and ongoing myocardial inflammation in recovered COVID-19 patients.

Huang^35^ 2020	China	Retrospective study	26 recovered patients	• 58% abnormal CMR • 54% myocardial edema • 31% LGE	Cardiac involvement after recovery from COVID-19	A proportion of recovered COVID-19 patients had cardiac involvement on CMR.

Rajpal^43^ 2020	USA	Case series	26 competitive athletes with mild COVID-19	• 15% CMR findings consistent with myocarditis • 46% LGE	Detect cardiac involvement through CMR in competitive athletes recovering from COVID-19	CMR may help stratify athletes recovering from COVID-19 as to risk of myocarditis.

Knight^44^ 2020	UK	Cross-sectional study	828 hospitalized patients positive for COVID-19 or with a clinical diagnosis	• 586 patients had elevated hsTNT • 51 underwent CMR: 69% of them had myocardial injury	Underlying cause of troponin elevation in COVID-19 infection	Myocardial injury detected by CMR is common in hospitalized COVID-19 patients.


In a multicenter CMR study evaluating 148 patients 2 months after being hospitalized with severe COVID-19 and elevated troponin levels, 54% of patients (80/148) had LGE and/or ischemia on CMR. The LGE pattern was inflammatory in 26% of patients, ischemic in 22%, and both in 6%.^42^ In contrast to Puntmann et al., no abnormalities on T1 or T2 mapping were seen compared with matched controls. More recently, another case-control study focused on healthcare workers free of known CVD, including 74 seropositive and 75 seronegative subjects. Of the total patients, only one was hospitalized briefly, while 11 (15%) were asymptomatic. The cohort underwent CMR and biomarker evaluation 6 months after enrollment. None of the end points—including left ventricular ejection fraction, indexed end-diastolic volume, LGE, global T1 and T2, and biomarkers such as NT-proBNP and troponin—were significantly different between the two groups.^38^

These studies highlight the importance of future research on cardiac involvement and its implications for recovered COVID-19 patients. However, larger multicenter studies with unified selection criteria and CMR imaging analysis are needed, and isolated T1/T2 abnormalities should be considered despite their uncertain relevance based on reduced biopsy specificity. While the incidence of cardiac involvement after COVID-19 may be lower than initially reported, it still carries critical long-term consequences given the extent of the pandemic. Healthy asymptomatic individuals who have a mild COVID-19 course appear unlikely to have significant cardiac involvement, but confirmatory studies are needed.

## Competitive Athletes and Return to Play

Early in the pandemic, there were significant concerns for the risk of myocarditis in otherwise healthy athletes with mild or no symptoms, leading to cessation of tournaments and discussions about cardiovascular screening prior to return to play after COVID-19 infection. This led to research studies evaluating the risk of SARS-COV-2 myocarditis or myocardial inflammation in athletes. In a retrospective study of 145 competitive student athletes recovering from mild-to-moderate COVID-19, only two patients (1.4%) had myocarditis.^39^ In a cohort study of 1,597 college athletes with CMR screening after COVID-19 infection, 37 athletes (2.3%) were diagnosed with clinical and subclinical myocarditis. However, there was significant variability in testing protocols and prevalence of myocarditis (eg, the prevalence was 0.31% based on symptom-based screening).^40^ In another study of 789 professional athletes with previous COVID-19 and mild or no symptoms, a screening strategy with troponin, electrocardiogram, and echocardiography identified 30 athletes (3.8%) with abnormalities. Only 5 athletes (0.6%) had CMR findings suggestive of myocardial inflammation (and they were restricted from play), while no adverse events occurred in those who resumed sports participation with negative screening.^41^

Currently, expert opinion considers cardiovascular testing to be unnecessary in those with no or mild COVID-19 symptoms after 10 days of exercise cessation and full resolution of symptoms. New cardiovascular symptoms or moderate or severe COVID-19 symptoms after recovery warrant a medical evaluation prior to return to sports.^45^

## Other Causes of Cardiac Injury in Covid-19

The following is a brief review of other potential etiologies that are covered elsewhere in this issue.

### Acute Coronary Syndrome

Acute respiratory infections, including viral and bacterial pneumonias, are well-recognized triggers for CVD and acute coronary syndrome (ACS).^46,47,48^ A recent study showed a 3- to 6-fold increase in the risk of myocardial infarction during the week after laboratory-confirmed infection with respiratory viruses (influenza virus, respiratory syncytial, etc.) compared with the risk during the year before or after infection.^49,50^

Admissions and care for patients with ACS were significantly impacted by COVID-19. A prospective international registry initiated early in the pandemic reported significant delays in patients seeking medical care and longer door-to-balloon times in COVID patients with ST elevation myocardial infarction (STEMI). There also were significantly higher rates of cardiogenic shock and quadrupling of in-hospital mortality compared with pre-COVID cohort databases.^51^ Another observational study of STEMI patients with concurrent SARS-COV-2 infection was suggestive of a higher thrombus burden and higher biomarkers levels (troponin T, D-Dimer, and C-reactive protein) compared with non–COVID-19 STEMI patients.^52^

### Heart Failure

Patients with heart failure (HF) who experience severe COVID-19 infection are particularly at risk for COVD-19–related morbidity and mortality.^19,53^ A cohort study of 132,000 HF patients admitted to the hospital for COVID-19 between April and September 2020 showed a 10-to-14-fold greater odds of dying versus patients with HF alone. Patients with HF and COVID-19 had more comorbidities and required more ICU stays, renal replacement therapy, and advanced cardiovascular monitoring. In addition, this cohort’s in-hospital mortality rate was almost 25% compared with 2.6% for HF patients without COVID-19 infection.^54^

Similar findings were shown in a retrospective study of 4,043 patients admitted for COVID-19 between March and May 2020. The 8.3% of patients with both COVID-19 and HF had a higher rate of cumulative in-hospital mortality compared with patients without HF (49% vs. 27%, *P* < .001) despite adjusting for age, body mass index, and comorbidities.^55^

Takotsubo cardiomyopathy has been described in the context of COVID-19 in several case reports and case series.^56,57,58,59^

## Conclusions

For almost 2 years, the world has faced a global pandemic due to COVID-19. Although vaccinations are reducing infection rates, the impact of the disease on global health has been massive. Cardiovascular comorbidities increase morbidity and mortality in COVID-19 patients, and the infection itself has been associated with myocardial injury and dysfunction. This can cause several complications, including myocarditis, arrhythmia, acute myocardial infarction, venous thromboembolic events, and heart failure. Therefore, to optimally manage COVID-19 patients, it is critical to not only assess their risk factors but also to be on alert for possible fatal cardiovascular complications. Additional studies with longer observation periods are needed to gain a deeper understanding of the potential long-term cardiovascular complications of COVID-19.

## Key Points

- Myocardial injury is common in hospitalized COVID-19 patients, particularly those with established cardiovascular disease.
- Myocardial injury can range from subclinical biomarker elevations to myocardial infarction, inflammatory cardiomyopathy, potential myocarditis, heart failure, and cardiogenic shock.
- The prevalence, implications, and optimal management of myocardial injury in nonhospitalized COVID-19 patients is a critical area for future research.
